# Impact of Charge-Trapping Effects on Reliability Instability in Al_x_Ga_1−x_N/GaN High-Electron-Mobility Transistors with Various Al Compositions

**DOI:** 10.3390/ma16124469

**Published:** 2023-06-19

**Authors:** Walid Amir, Surajit Chakraborty, Hyuk-Min Kwon, Tae-Woo Kim

**Affiliations:** 1 Department of Electrical, Electronic and Computer Engineering, University of Ulsan, Ulsan 44610, Republic of Korea; 2 Department of Semiconductor Processing Equipment, Semiconductor Convergence Campus, Korea Polytechnics, Anseong-si 17550, Republic of Korea

**Keywords:** AlGaN/GaN HEMT, interfacial degradation, fast-transient charge-trapping, pulsed I–V, constant voltage stress (CVS), threshold voltage degradation (∆*V_T_*), 1/*f* low-frequency noise, volume trap density (*N_t_*)

## Abstract

In this study, we present a detailed analysis of trapping characteristics at the Al_x_Ga_1−x_N/GaN interface of Al_x_Ga_1−x_N/GaN high-electron-mobility transistors (HEMTs) with reliability assessments, demonstrating how the composition of the Al in the Al_x_Ga_1−x_N barrier impacts the performance of the device. Reliability instability assessment in two different Al_x_Ga_1−x_N/GaN HEMTs [x = 0.25, 0.45] using a single-pulse *I_D_–V_D_* characterization technique revealed higher drain-current degradation (∆*I_D_*) with pulse time for Al_0.45_Ga_0.55_N/GaN devices which correlates to the fast-transient charge-trapping in the defect sites near the interface of Al_x_Ga_1−x_N/GaN. Constant voltage stress (CVS) measurement was used to analyze the charge-trapping phenomena of the channel carriers for long-term reliability testing. Al_0.45_Ga_0.55_N/GaN devices exhibited higher-threshold voltage shifting (∆*V_T_*) caused by stress electric fields, verifying the interfacial deterioration phenomenon. Defect sites near the interface of the AlGaN barrier responded to the stress electric fields and captured channel electrons—resulting in these charging effects that could be partially reversed using recovery voltages. The quantitative extraction of volume trap density (*N_t_*) using 1/*f* low-frequency noise characterizations unveiled a 40% reduced *N_t_* for the Al_0.25_Ga_0.75_N/GaN device, further verifying the higher trapping phenomena in the Al_0.45_Ga_0.55_N barrier caused by the rougher Al_0.45_Ga_0.55_N/GaN interface.

## 1. Introduction

High-electron-mobility transistors (HEMTs) based on III-V materials have been the next generation of high-power, high-radio-frequency, and high-temperature devices because of their high carrier concentration, high carrier mobility, and high breakdown voltage [1,2,3]. GaN-based HEMTs have recently attracted much attention because of their remarkable material properties and device performances, notably in high-power and RF applications up to the sub-terahertz regime [4,5,6]. These advantageous properties and performances are caused primarily by the excellent quality of the epitaxial layer consisting of the Al_x_Ga_1−x_N barrier and the GaN channel layer, resulting from the fundamental electronic properties of two-dimensional electron gas (2DEG) on top of Si, Sapphire, and silicon carbide (SiC) substrates [7,8,9]. Because high-density 2DEG accumulates at the Al_x_Ga_1−x_N/GaN interface, those electronic properties would reflect the quality of the interface via scattering procedures caused by dislocations [10]. During device operation, the interface quality of the Al_x_Ga_1−x_N/GaN is essential for improving carrier transport in the channel [10].

Reliability concerns of Al_x_Ga_1−x_N/GaN HEMTs have been caused by trap effects related to a drain, gate lag, and current collapse with various types of degradation [11,12,13]. The AlGaN layer, which is usually the surface layer and has an interface with the GaN channel, is a source of reliability instability, including trapping in the gate-to-drain access region, deep-level, and Al_x_Ga_1−x_N/GaN interface [14]. Although most surface traps can be passivated with different kinds of passivation layers (e.g., SiN_x_, SiO_2_, and Al_2_O_3_), optimization of the traps inside the Al_x_Ga_1−x_N layer and the Al_x_Ga_1−x_N/GaN interface is still an ongoing investigation. The reliability instability issues of the Al_x_Ga_1−x_N/GaN HEMTs are worse than that of conventional *Si*-based devices because of the interface quality of the Al_x_Ga_1−x_N/GaN [15,16,17].

**Figure 1:**
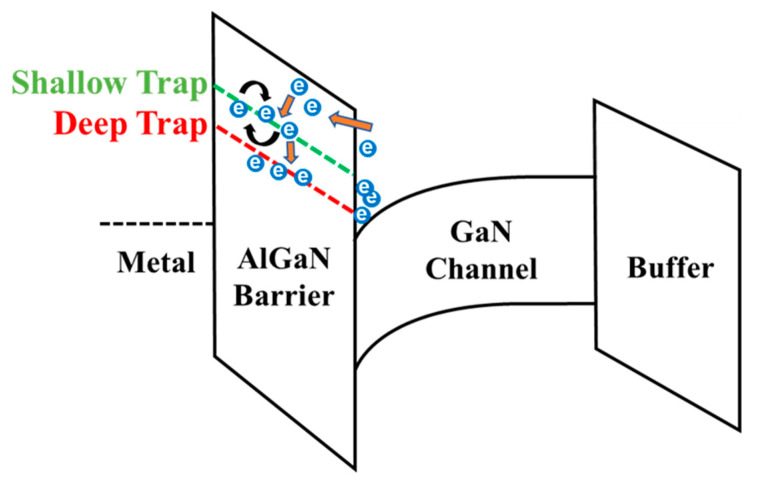
Schematic of the band diagram of Al_x_Ga_1−x_N/GaN HEMTs defining the “Shallow” and “Deep” trap states that capture tunneling channel carriers.

The defect sites in the AlGaN barrier layer and the interface Al_x_Ga_1−x_N/GaN are the predominant cause of the transient-charging effects (Figure 1) [18,19]. The transient-charging effects follow two different processes, fast and slow transient charging. Channel carriers are easily injected into shallow defects (fast-transient charging) in the Al_x_Ga_1−x_N barrier layer and the interface of Al_x_Ga_1−x_N/GaN. Then, trapped charges in the shallow trap site follow thermally activated electron migration via trap-to-trap conduction (slow transient charging). The fast-transient-charging effect is responsible for mobility degradation and threshold voltage (*V_T_*) instability in AlGaN/GaN HEMTs, while the slow transient charging causes long-term stress *V_T_* instability. All of these are major concerns for implementing GaN-based HEMTs in future applications. Improving the reliability instability of Al_x_Ga_1−x_N/GaN HEMTs requires thoroughly analyzing the trapping effects because the channel carriers can easily tunnel into the pre-existing defect sites in the Al_x_Ga_1−x_N barrier layer and the interface Al_x_Ga_1−x_N/GaN.

In this study, a comprehensive analysis of the trapping effects in Al_x_Ga_1−x_N/GaN HEMTs with varying Al compositions was performed to optimize the device structure to obtain improved performance. We performed a reliability instability assessment in two different Al_x_Ga_1−x_N/GaN HEMTs [x = 0.25, 0.45] using a single-pulse *I_D_–V_D_* technique that could be demonstrated by fast-transient-charging effects. For long-term reliability testing, we performed constant voltage stress (CVS) measurements under high-drain bias conditions to analyze the charge-trapping phenomena of the channel carriers. *V_T_* shifting during constant voltage stress was compared between the devices to verify the interfacial degradation phenomena. Furthermore, the flicker noise characteristics were analyzed to gain knowledge of the dominant defect locations of the two structures. Finally, to verify the quantitative analysis of the trapping effects, the trap density (*N_t_*) of both samples was calculated using the carrier mobility fluctuation (CMF) model [20].

## 2. Experimental Details

**Figure 2:**
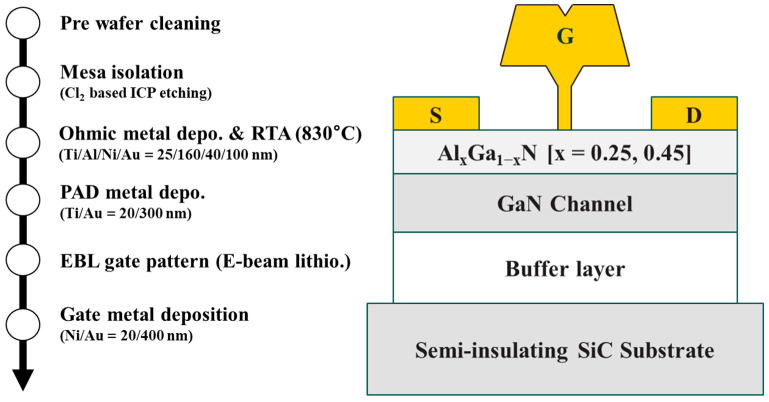
Fabrication process flow and the cross-section illustration of the Al_x_Ga_1−x_N/GaN HEMTs with different Al [25%, 45%] compositions in the barrier layer.

Figure 2 represents the fabrication process flow and the cross-sectional illustration of the Al_x_Ga_1−x_N/GaN HEMTs used in this study. The epitaxial layers were grown on a semi-insulating SiC substrate using metal-organic chemical vapor deposition (MOCVD). Each layer was grown in the following order: ~270 nm of an AlN buffer layer, ~400 nm of GaN channel, and ~20 nm of an Al_x_Ga_1−x_N [x = 0.25, 0.45] barrier layer. Cl_2_-based inductively-coupled plasma (ICP) etching was used to isolate the devices for the mesa isolation procedure. The substrate was then cleaned for 30 s with a 1:5 solution of HCl and deionized water to remove any native oxide. The ohmic metallization of the source and drain was performed by an e-beam evaporator with a metal scheme of Ti/Al/Ni/Au (25/160/40/100 nm). Rapid thermal annealing was used to alloy the ohmic contacts at 830 °C and under N_2_ ambient for 30 s. An additional padding layer of Ti/Au (20/300 nm) was deposited by an e-beam evaporator to ensure proper probe contact during device characterization. The contact resistance (*R_C_*) and sheet resistance (*R_SH_*) from TLM measurements were 0.25 Ω·mm and 380 Ω/□ for the Al = 25% sample and 0.28 Ω·mm and 420 Ω/□ for the Al = 45% sample, respectively. Finally, the gate pattern was defined using e-beam lithography, and a T-shaped Ni/Au (20/400 nm) short-channel gate was deposited. Gate-source and gate-drain distances were kept symmetrical, and the drain-to-source distance was fixed at 2 µm. All electrical characteristics were analyzed using the Keysight B1500A semiconductor parameter analyzer. The fast-transient-charging effect characterization was conducted using the single-pulse *I_D_–V_D_* with a pair of B1530A waveform generator modules. For the *1/f* low-frequency flicker noise measurements, we used a dynamic signal analyzer HP 35670A and a current preamplifier SR570.

## 3. Results and Discussion

### 3.1. Charge-Trapping Analysis with Pulsed I–V

**Figure 3:**
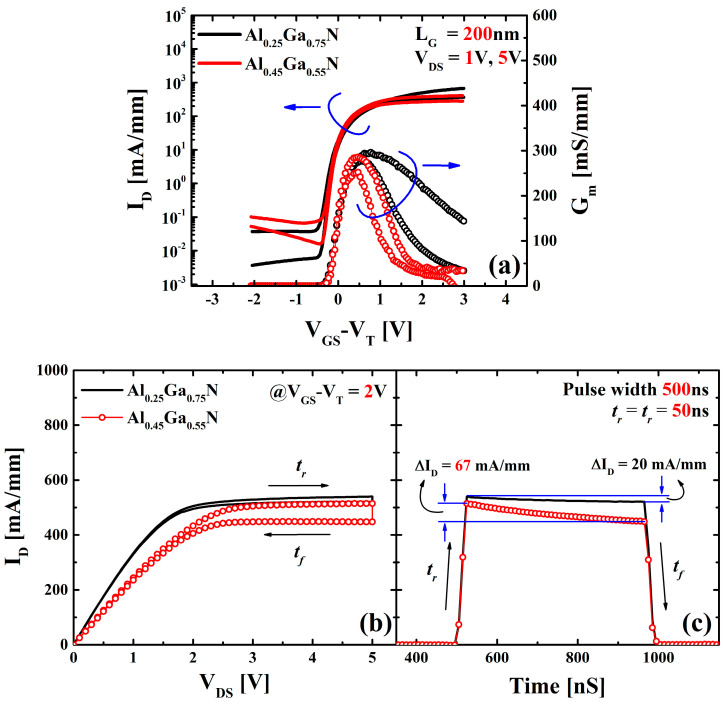
(**a**) DC transfer characteristics of the samples at *V_DS_* = 1, 5 V with respect to the gate overdrive voltage (*V_GS_*–*V_T_*). (**b**) A single-pulse *I_D_–V_D_* characteristics of AlGaN/GaN HEMTs with different Al compositions. (**c**) Rapid deterioration of the drain current over time when a maximum pulse is applied to both gate and drain, which is consistent with the pulsed *I_D_–V_D_* sweep.

Figure 3a shows the DC transfer characteristics comparison of the devices with respect to the gate overdrive voltage (*V_GS_*–*V_T_*). Although the device characteristics are quite similar in DC measurements, the Al = 25% sample showed slightly higher drain current *I_D_* (at high *V_GS_*–*V_T_*) and transconductance *G_m_*. Figure 3b illustrates single-pulse *I_D_−V_D_* characteristics with different Al compositions in the barrier layer. The output characteristics of a single-pulse *I_D_–V_D_* technique with the rise (*t_r_*) and fall time (*t_f_*) of 50 ns were measured with a *V_D_* sweep. Rise and fall times were kept small to achieve trap-free *I_D_–V_D_* characteristics [21]. A short pulse width of the gate and drain was applied during the measurement, reducing fast-transient trapping/de-trapping effects. 

A significant reduction in the drain-current (∆*I_D_*) is observed during the fall-down trace for the Al = 45% sample compared with the Al = 25% sample, related to the filling of the resonant traps during the rise time and pulse width through the fast-transient charging process. DC measurements cause a significant degradation because of higher integration time (~5 ms) [22,23].

Figure 3c depicts the fast degradation in the drain-current with respect to time when the gate pulse is *V_GS_–V_T_* = 2 V and drain bias is *V_DS_* = 5 V, corresponding to the pulsed *I_D_−V_D_* characteristics. Channel carriers are trapped in the trap states near the interface of the Al_x_Ga_1−x_N barrier layer and the interface Al_x_Ga_1−x_N/GaN [23,24]. *I_D_* degradation for Al_0.25_Ga_0.75_N/GaN device during 500 ns pulse width is ~20 mA/mm, while Al_0.45_Ga_0.55_N/GaN device illustrates *I_D_* degradation in ~67 mA/mm. A significantly higher *I_D_* degradation for the Al = 45% sample corresponds to a rougher interface between the barrier and GaN channel caused by higher lattice mismatching.

Drain-current degradation with respect to pulsed time is related to charge-trapping in the defect sites, which can be explained by the model of charging processes [25]. Channel carriers can be tunneled into the shallow defect sites in the AlGaN barrier layer and can occur to thermally activated electron migration between the defect sites with temperature dependency. The location of these defect sites is below the conduction band, as illustrated in Figure 1. Because of the extremely low trap energy of these shallow traps and the high density of states (DOE) from the GaN conduction band, the charging process will have a fast charging time. Slow transient charging can be attributed to the capture of secondary electrons induced from the trapped charges from the fast charging process.

### 3.2. Charge-Trapping Analysis with Constant Voltage Stress Condition

**Figure 4:**
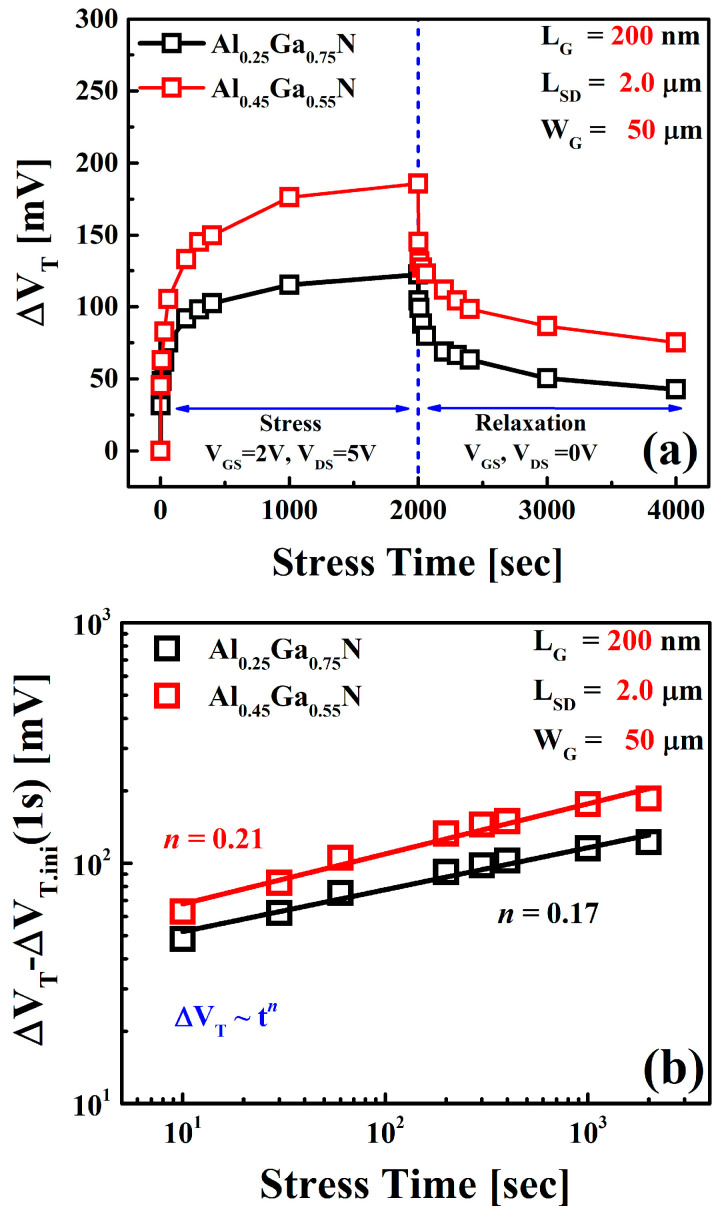
(**a**) Threshold voltage shift (∆*V_T_*) characteristics of AlGaN/GaN HEMTs during constant voltage stress at high drain bias (*V_DS_* = 5 V) condition illustrating charge-trapping and de-trapping properties of the channel electrons. (**b**) Power-law time dependency of the observed Δ*V_T_* excluding the fast-transient charge-trapping components (Δ*V_T_*−Δ*V_T.initial_*_(1 s)_) in two samples.

A long-term reliability evaluation was performed under high electric field conditions to verify the interfacial degradation from charge-trapping. Figure 4a illustrates the charge-trapping and de-trapping characteristics of two samples during a complete cycle of constant voltage stress at both gate and drain and relaxation cycle. Applied stress conditions were *V_GS_* = 2 V and *V_DS_* = 5 V. Threshold voltage shifting (∆*V_T_*) from trapping in the interface states was evident. Channel carriers are trapped in the defect sites of the Al_x_Ga_1−x_N barrier via the interface caused by a high electric field and thin barrier layer [24,26]. The degradation in *V_T_* is consistent with the electron trapping at the Al_x_Ga_1−x_N barrier layer defect locations from the GaN channel layer. This trapping phenomenon can be partially recovered by applying recovery voltages of *V_GS_* and *V_DS_* = 0 V. 

The fast-transient trapping effect, which is active during a short (<1 ms), is accountable for the substantial change in the initial *V_T_* (1 s). This effect is caused by the tunneling of channel carriers in the pre-existing defect sites inside the Al_x_Ga_1−x_N barrier. The ∆*V_T_* characteristics at Al = 45% had a higher initial ∆*V_T_* and more degradation than at Al = 25%.

The time dependence of the *V_T_* was investigated to quantify the charge-trapping phenomenon (Figure 4b). The fast-transient charge-trapping component, which is supposed to saturate fully after 1 s of stress, may be eliminated, and the power-law equation can be used to describe the time dependence ∆*V_T_*~*t^n^* of the ∆*V_T_* (∆*V_T_* − ∆*V_T.initial_* (1 s)) [23,27,28]. Both devices degrade according to the power-law kinetics. Time exponent, *n*, is in the range of 0.17–0.21, a similar but somewhat lower range than for the Al = 25% device, corresponding to a lower interfacial degradation [29]. Regardless of the value of *n*, the ∆*V_T_* values of Al = 45% devices are much higher than the Al = 25% device associated with higher trap states in the Al_0.45_Ga_0.55_N barrier.

### 3.3. Quantitative Analysis of Trap Density with 1/f Low-Frequency Noise

Low-frequency noise (LFN) is an effective tool for analyzing the interface states in a semiconductor device—predominantly responsible for performance degradation. A flicker noise(*1/f* noise) is usually generated from the following two causes: Carrier Number Fluctuation (CNF) and Carrier Mobility Fluctuation (CMF) [20]. Both are related to charge-trapping from the channel to the gate dielectric or barrier layer. Carrier interaction between the channel and the near-interface dielectric/barrier traps causes CNF noise. These charging effects also cause fluctuation in carrier mobility and result in correlated mobility fluctuations [30,31]. Both CNF and CMF should be considered for the quantitative trap extraction to evaluate accurate charge-trapping phenomena. With the CMF model proposed in the literature [20], it is possible to gain knowledge on the *1/f* noise in all the operation regions (from linear to saturation and weak to strong inversion).

**Figure 5:**
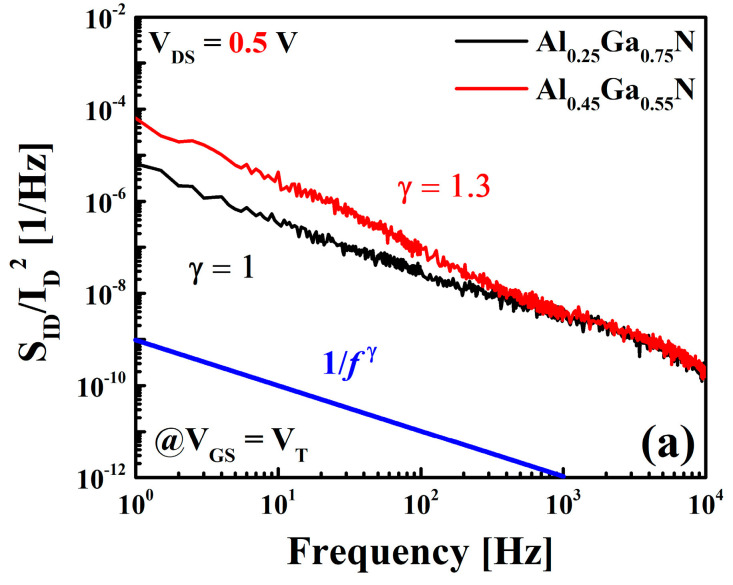

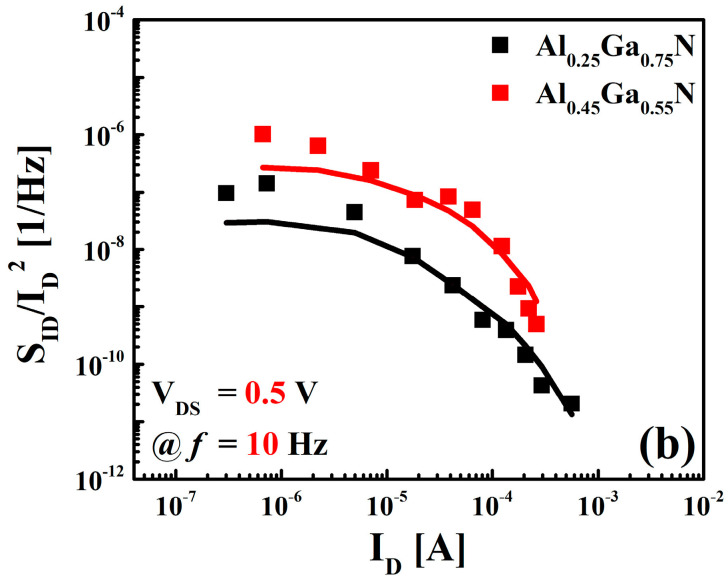
(**a**) Comparison of the normalized drain-current power spectral density (PSD) (*S_ID_/I_D_*^2^) at *V_GS_ = V_T_* and *V_DS_* = 0.5 V. The frequency component (ℽ) is in the range of 1~1.3. (**b**) Fitting curves of *S_ID_/I_D_*^2^ values using the CMF model calculated at a frequency of 10 Hz.

The *1/f* noise measurements were performed from 1 Hz to 10 kHz at a fixed drain bias of *V_DS_* = 0.5 V from off-state to accumulation, including the linear region. Figure 5a illustrates the normalized power spectral density (*S_ID_*/*I_D_^2^*) with respect to the frequency at *V_GS_* = *V_T_* condition. The power-law equation (1/*f^ℽ^* function) is used to explain the frequency dependency of power spectral density (PSD) [18]. The 1/*f^ℽ^* function was fitted with the measured data over the frequency range of 1 Hz to 10 kHz to extract the value of the frequency component (ℽ) [19]. Based on Table 1, the value of ℽ is in the range of 1–1.3 (near 1), indicating that the defects/traps had uniform depth and energy [32]. Al = 45% devices had a ℽ value of 1.3 (Over 1), indicating that the most dominant trap locations are close to the interface of the Al_0.45_Ga_0.55_N barrier and GaN channel.

For the quantitative analysis of the trap states, the trap density (*N_t_*) was extracted using the CMF model, as represented by [20,31]:

(1) $$\frac{S_{ID}}{I_{D}^{2}}={\left(\frac{G_{m}}{I_{D}}\right)}^{2}{\left(1+\alpha_{SC}\mu_{eff}C_{B}\frac{I_{D}}{G_{m}}\right)}^{2}S_{Vfb}$$

where the variable *α_sc_* is the coefficient for Coulomb scattering, *µ_eff_* is the effective mobility of the carriers, and *C_B_* is the capacitance per unit area of the AlGaN barrier. *S_Vfb_* is the flat-band voltage and can be defined as follows [33,34,35]:

(2) $$S_{Vfb}=\frac{q^{2}kT\lambda N_{t}}{WLC_{B}^{2}f}$$

where *q*, *kT*, and *N_t_* are symbols used to represent elemental charge, thermal energy, and trap density, respectively. *λ* = [4π(2m*Φ_B_)^1/2^/*h*]^−1^ represents the attenuation tunneling distance (*Φ_B_* denotes the barrier height) [36]. Figure 5b illustrates a good fitting between the normalized drain-current power spectral density (*S_ID_*/*I_D_*^2^) and the right side of Equation (1), which prevails in the CMF model. Using Equation (2) and the *S_Vfb_* extracted from the fitting, *N_t_* for both devices was extracted. Al = 45% devices had a 40% higher *N_t_* value of 3 × 10^19^ cm^−3^·eV^−1^ compared with 1.8 × 10^19^ cm^−3^·eV^−1^ for the Al = 25% devices. The reason for these noise characteristics is attributed to the fact that the Al_0.45_Ga_0.55_N/GaN interface creates higher defect sites near the interface, which increases the probability of the channel electron tunneling into the AlGaN barrier layer.

## 4. Conclusions

We demonstrated an in-depth trapping characteristic analysis of the Al_x_Ga_1−x_N/GaN interface of AlGaN/GaN HEMTs based on the Al composition in the Al_x_Ga_1−x_N barrier and how it affects device performance. Higher *I_D_* degradation for the Al_0.45_Ga_0.55_N/GaN devices during the pulsed *I_D_*−*V_D_* characterization was attributed to the higher fast-transient trapping in the Al_0.45_Ga_0.55_N/GaN interface and reliability instability. During constant voltage stress conditions, the Al_0.45_Ga_0.55_N/GaN device had a higher *V_T_* shift corresponding to higher trapping in the Al_0.45_Ga_0.55_N barrier. A larger time exponent *n* in the Al_0.45_Ga_0.55_N/GaN device indicated higher interfacial degradation. During quantitative extraction of volume trap density, the Al_0.45_Ga_0.55_N/GaN device had a 40% higher *N_t_*, further verifying the higher trapping phenomena in the Al_0.45_Ga_0.55_N barrier caused by the rougher Al_0.45_Ga_0.55_N/GaN interface. These results demonstrate that trapping effects, which impact device performance considerably, are influenced primarily by the quality of the interface between the AlGaN and GaN layers. Future applications of the GaN HEMT devices can benefit from enhanced device properties by lowering the Al content to reduce lattice mismatches.

## Figures and Tables

**Table 1:** Comparison of the key reliability parameters of the AlGaN/GaN HEMTs.

Sample	∆*I_D_* [mA/mm]	*n*	ℽ	*N_t_* [cm^−3^·eV^−1^]
Al_0.25_Ga_0.75_N/GaN	20	0.17	1	1.8 × 10^19^
Al_0.45_Ga_0.55_N/GaN	67	0.21	1.3	3 × 10^19^

## Data Availability

The data presented in this study are available on request from the corresponding author.
